# Oral microbiome and mycobiome dynamics in cancer therapy-induced oral mucositis

**DOI:** 10.1038/s41597-025-04671-z

**Published:** 2025-03-20

**Authors:** Laurentia Nodit, Joseph R. Kelley, Timothy J. Panella, Antje Bruckbauer, Paul G. Nodit, Grace A. Shope, Kellie Peyton, Dawn M. Klingeman, Russell Zaretzki, Alyssa Carrell, Mircea Podar

**Affiliations:** 1 University of Tennessee Graduate School of Medicine Knoxville, Department of Pathology and Biorepository, Knoxville, TN USA; 2 University of Tennessee Graduate School of Medicine Knoxville, Department of Radiation Oncology, Knoxville, TN USA; 3 Genesis Care of North Carolina, Asheville, NC USA; 4 University of Tennessee Graduate School of Medicine Knoxville, University Cancer Specialists, Knoxville, TN USA; 5 University of Tennessee Health Science Canter, Graduate School of Medicine, Memphis, TN USA; 6 Oak Ridge National Laboratory, Biosciences Division, Oak Ridge, TN USA; 7 University of Tennessee Business Analytics and Statistics, Knoxville, TN USA

**Keywords:** Oral cancer, Oral cancer

## Abstract

Cancer therapy-induced oral mucositis is a frequent major oncological problem, secondary to cytotoxicity of chemo-radiation treatment. Oral mucositis commonly occurs 7–10 days after initiation of therapy; it is a dose-limiting side effect causing significant pain, eating difficulty, need for parenteral nutrition and a rise of infections. The pathobiology derives from complex interactions between the epithelial component, inflammation, and the oral microbiome. Our longitudinal study analysed the dynamics of the oral microbiome (bacteria and fungi) in nineteen patients undergoing chemo-radiation therapy for oral and oropharyngeal squamous cell carcinoma as compared to healthy volunteers. The microbiome was characterized in multiple oral sample types using rRNA and ITS sequence amplicons and followed the treatment regimens. Microbial taxonomic diversity and relative abundance may be correlated with disease state, type of treatment and responses. Identification of microbial-host interactions could lead to further therapeutic interventions of mucositis to re-establish normal flora and promote patients’ health. Data presented here could enhance, complement and diversify other studies that link microbiomes to oral disease, prophylactics, treatments, and outcome.

## Background & Summary

Cancer-therapy induced mucositis (CTOM) is a major complication of anti-cancer treatments and has been the focus of numerous studies to identify pathophysiological elements and derive appropriate responses^1–7^. Among the keystone drivers in CTOM development and severity are direct epithelial cytotoxicity, leading to apoptosis and atrophy, followed by dysfunction of the epithelial barrier with endotoxin and bacterial translocation through tight mucosal junctions, subsequent innate immune activation and up-regulation of the inflammatory reaction^6^. The host-microbe interactions and dynamic changes in resident microbial composition (bacteria and fungi) during development of oral CTOM has also been recognized^8–15^. The development of appropriate anti-microbial therapy is difficult^16^, hence the need to better understand the role of the oral microbiota in the development of CTOM. While most microbial species cause no harm under healthy conditions, the disruption of the delicate hemostasis between host defense and the commensal microbiome contribute to CTOM^17^. Next generation sequencing (NGS) approaches (amplicons and shotgun metagenomic sequencing) along with informatics and statistical advances have made it feasible to analyze the diversity and relative abundance of individual microbial taxa, as well as their physiological potential across a wide range of human populations and clinical studies^18–21^.

In this study we employed amplicon sequencing to examine the dynamics of bacterial and fungal communities prior, during and after therapy for oral squamous cell carcinoma. Longitudinal sampling captured the development of mucositis stages and the post treatment amelioration of symptoms in affected patients. The primers targeted the bacterial small subunit ribosomal RNA (16S rRNA) genes and the fungal ribosomal internal transcribed spacer (ITS), respectively.

## Methods

### Cohort study design

The cohort for the longitudinal study included nineteen patients with biopsy-confirmed oral or oropharyngeal squamous cell carcinoma (SCC) and eleven healthy control volunteers, most of which recruited from first-degree relatives of the enrolled cancer patients. The hospital oncology team treated all patients with resection surgery, chemo- or radiation therapy as deemed appropriate, and all participants had a dental evaluation prior to sample collection. Individual clinical information is summarized in Table 1 and further detailed in the Supplementary Table. Excluded from the study were patients with squamous cell carcinoma of larynx or hypopharynx. All patients received both chemotherapy (high dose Cisplatin, weekly Cisplatin, Carbo/Taxol or Carboplatin/ 5- fluorouracil) and radiotherapy (in tumour bed or neck), except for one patient who underwent radiation therapy only. Not included in the study were patients receiving immunotherapy or stereotactic body radiation. The study (recruitment, sample collection and data sharing) was approved by the University of Tennessee Health Science Center Knoxville IRB (study number 4652) and all participants provided written informed consent.

**Table 1:** Subjects and associated metadata.

Cancer	Age	Sex	Stage	P16	Surgery	Radiation	Chemotherapy	Dentition	Galera	Control	Age	Sex
Oral Cavity										OM-3	61	F
OM-1	61	M	IVa, (T4, N0, M0)	+	Unresectable	70 Gy	Carbo/ Tax	Poor	No	OM-4	56	F
OM-2	55	M	IVA (T4a, N0, M0)	−	Yes Margin (−)	60 Gy	Cis 100 Q3wk	Excellent	No	OM-5	24	F
OM-14	75	F	II (T2 NX M0)	−	Yes Margin (+)	70 Gy	Carbo/ Tax	Poor	No	OM-6	51	F
OM-20	73	F	III (T3, N0, M0)	−	Yes Margin (+)	70 Gy	Cis 40 Weekly	Excellent	Yes	OM-12	85	M
OM-22	62	F	IVA (T2, N2b, M0)	−	Yes Margin (−)	60 Gy	Cis 100 Q3wk	Excellent	Yes	OM-15	73	M
OM-26	52	M	III (T2, N1 M0)	−	Yes Margin (−)	60 Gy	Carbo/ 5FU	Excellent	No	OM-18	58	F
OM-34	75	F	IVA (T4, N2b, M0)	−	Yes Margin (−)	60 Gy	Cis 40 Weekly	Poor	No	OM-23	65	M
OM-9	78	F	II(T2, N0, M0)	−	Yes Margin (−)	60 Gy	None	Excellent	No	OM-25	60	F
Oro-Pharynx										OM-27	60	F
OM-10	72	F	I (TX, N1, M0)	+	No	70 Gy	Carbo/ Tax	Excellent	No	OM-30	72	M
OM-11	80	F	I (T2, N1 M0)	+	No	70 Gy	Carbo/ Tax	Excellent	No	OM-32	48	M
OM-13	47	M	IVa (T2, N2c M0)	−	No	70 Gy	Cis 40 Weekly	Poor	No			
OM-16	61	M	I (T1, N1, M0)	+	Yes	70 Gy	Cis 100 Q3wk	Excellent	Yes			
OM-17	36	M	IVa (T3, N2b M0)	−	No	70 Gy	Cis 100 Q3wk	Poor	No			
OM-19	62	M	I (T1, N1 M0)	+	No	70 Gy	Cis 100 Q3wk	Poor	No			
OM-24	59	M	II (T3, N1, M0)	+	Yes Margin (+/−)	66 Gy	Cis 40 Weekly	Excellent	Yes			
OM-28	50	M	II (T2, N2, M0)	+	No	70 Gy	Cis 100 Q3wk	Excellent	Yes			
OM-29	73	M	I (T2, N1, M0)	+	No	70 Gy	Cis 40 Weekly	Excellent	No			
OM-31	72	M	I (T1, N1, M0)	+	Yes	70 Gy	Cis 40 Weekly	Excellent	No			
OM-33	63	M	I (T0, N1, M0)	+	Yes	70 Gy	Carbo/ Tax	Excellent	No			

Subjects included in the study. Cancer stage classification is according to AJCC Cancer Staging Manual^28^. P16 state was determined using standard immunohistochemistry for P16, a surrogate marker for oncogenic HPV infection. Surgical resection was performed when feasible and included partial glossectomy, floor of mouth resection, mandibular resection and cervical lymph nodes dissection, tonsillectomy, and selective cervical lymphadenectomy based on each tumour site of origin and extent of involvement. Radiation therapy was performed in all patients as intensity-modulated radiation therapy (IMRT). The prescribed dose was 70 Gy in 35 fractions for most cases, with lower doses (60 Gy in 30 fractions or 66 Gy in 33 fractions) applied in selected cases, based on the patient’s tolerability. Adjuvant chemotherapy was performed in all, but one patient and followed one of the following four regimens: high dose cisplatin (Cisplatin 100 mg/m^2^ given once every 3 weeks), low dose cisplatin (Cisplatin 40 mg/m^2^ once a week), Carbo/Tax regimen (weekly Carboplatin and paclitaxel (Taxol) administration) or Carbo/5Fu (Carboplatin and 5-Fluorouracil). Excellent dentition was classified as healthy appearing gums and teeth with no visible cavities of decay. Poor dentition was significant gum recession, grossly visible cavities or unrestorable decayed teeth. Additional metadata is presented in the Supplementary Tables S1, S2.

#### Oral cavity squamous cell carcinoma subjects

Eight patients with oral cavity SCC (three males and five females, average age of 65) were enrolled (Table 1 and Supplementary Table S1). One patient was deemed medically unresectable, while the other patients were all treated with definitive surgery. Patients received adjuvant radiation therapy using intensity-modulated radiation therapy (IMRT) with 6MV photons to the oral cavity and nodal basins at 2Gray per fraction to 50 Gy. The primary site was treated using a sequential boost to 60 Gy in five patients and to 70 Gy in two patients with a positive margin. All but one patient received concurrent chemotherapy.

#### Oro-pharyngeal squamous cell carcinoma subjects

Eleven patients with SCC of the oropharynx (nine males and two females, median age of 60) were enrolled (Table 1). Among them, 9 were p16 positive (82%). All patients were treated with IMRT with standard fractionation. One patient with a positive margin after resection was treated to 66 Gy with all remaining oropharynx patients were treated to 70 Gy. All oropharyngeal patients received concurrent chemotherapy.

#### Galera ROMAN clinical trial

Five of the cancer patients (OM16, OM 20, OM22, OM24, and OM28) were concomitantly enrolled on the Galera ROMAN phase 3 clinical trial^22^ and randomized to placebo or Avasopasem infusion daily one hour prior to radiation therapy. All patients enrolled on the Galera trial were treated with standard of care surgery, chemotherapy, and radiation as determined by their physicians, and were not given other mouthwash formulations. The physicians in the study were blinded to treatment arms of the trial.

#### Control subjects

Cancer-free control subjects were recruited from relatives of enrolled patients and from the general population. They included 4 males and 7 females between the ages of 24–73 years (Table 1 and Supplementary Table S2). Exclusion criteria were recent (within the past month) administration of antibiotics.

#### Patient assessment

Patients were evaluated weekly for the development of oral mucositis based on the World Health Organization (WHO) Oral Mucositis Assessment Scale^23^ and the Radiation Therapy Oncology Group (RTOG) Mucositis Assessment Scale. Grade 0 mucositis was reserved for patients with no significant oral findings. Patients with soreness/erythema were classified as grade 1. The presence of ulcers with preservation of ability to eat solid food was described as grade 2, whereas ulcers that required liquid diet only was classified as grade 3. Grade 4 mucositis was diagnosed when alimentation was not possible. The patients’ diet was recorded each week as solid food, soft food, liquids only, or NPO (all nutrition provided through a feeding tube). The presence or absence of thrush was recorded each week along with the use of any antibiotics, steroids and mouthwash (see Supplementary Table S1 for associated metadata).

### Sample collection and processing

**Fig. 1:**
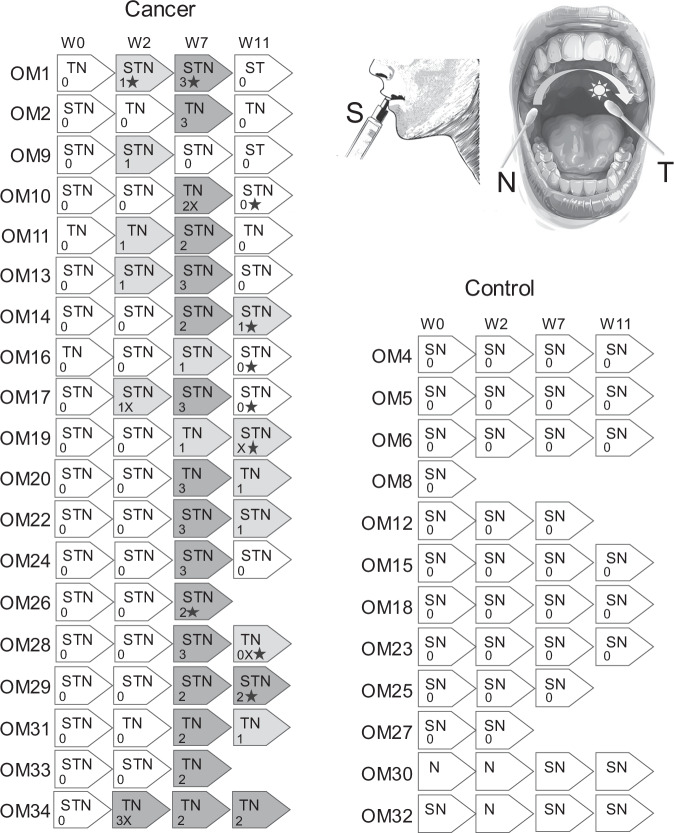
Longitudinal sampling design for control and cancer subjects. Saliva (S), oral mucosa swab samples from tumour (T) or normal site (N) were collected at weeks 0, 2, 7 and 11. The number indicate absence (0, no shading) or presence (shaded) of mucositis of various grades (1,2,3) and/or thrush (x). Administration of anti-fungal medication is indicated by the star symbols. The cartoons summarize the type of collected samples.

Unstimulated whole saliva samples and mucosal swabs were collected to evaluate the oral cavity microbiome. Initial samples were collected 7–10 days prior to the initiation of radiation or chemo-radiation treatments. Additional samples were collected again during the third week of radiation therapy (12–15 Gy) at a time point expected to coincide with mild mucositis. A third round of samples was collected between a dose of 60–70 Gy to correspond with severe mucositis stage. A final sample was collected one month after completing therapy. Samples were collected from healthy control participants at similar four time points as the SCC cancer patients to examine normal variations occurring in the oral cavity microbiome of healthy participants over time. Some samples could not be collected from some subjects. A schema of the sampling strategy is presented in Fig. 1.

All subjects provided 1 ml of saliva by passive drool into a cryovial using a Saliva Collection Aid (Salimetrics LLC, State College, PA). However, due to xerostomia that develops during radiation therapy collection of saliva was not possible in some patients during the third week of therapy and in most patients during the final week of radiation. Sterile rayon-tipped swabs (Copan Diagnostics Inc, CA, USA) were used to sample the mucosa or site of mucositis within the oral cavity or oropharynx by rolling/rubbing the swab over the affected surface. In cancer patients, one sample was collected from the tumour site or resection bed at each time point and a second sample was collected from the contralateral oral cavity or oropharynx. In healthy volunteers only a single swab was collected from the entire buccal mucosa at each time point. Swabs were immediately preserved in 1 ml of S1 Lysis buffer (PureLink™ Microbiome DNA Purification Kit, Invitrogen, Thermo Fisher Scientific, Waltham, MA). Swabs and saliva were transferred to the University of Tennessee Medical Canter Biorepository and stored at −80 °C until further processing. All participants and retrieved specimens were assigned a unique code in the biorepository. Only de-identified specimens were used for DNA extraction, amplicon sequencing, and data analyses.

For DNA extraction we used the PureLink Microbiome DNA Purification Kit following the manufacturers’ protocol for saliva and buccal swaps. Either 200 µl of saliva mixed with 600 µl of S1 lysis buffer or 800 µl of lysis buffer from preserved swabs were used. DNA concentration was determined using the Qubit dsDNA BR Assay kit (Life Technologies, Thermo Scientific Inc, Waltham, MA) with a Qubit 4 Fluorometer (Invitrogen, Thermo Fisher Scientific, Waltham, MA). Extracted DNA was aliquoted and stored at −80 °C.

### Amplicon library preparation and sequencing

To generate the amplicon libraries, we used the Zymo Quick-16S™ NGS Library Prep Kit (Zymo Research Corporation, Irvine, CA, USA) with the multi-step Real Time Polymerase Chain Reaction approach, following manufacturer’s instructions. In the first step, Targeted Sequence Amplification was performed using a forward and reverse primer mix that targets the V4 variable region of the 16S rRNA gene of Archaea and Bacteria (515FYM: 5′GTGYCAGCMGCCGCGGTAA; 515F_TM7:5′GTGCCAGCMGCCGCGGTCA; 515Propioni: 5′ GTGCCAGCAGCCGCGGTGA; 806 R: 5′GGACTACNVGGGTWTCTAAT; 806R_Propioni: 5′GGACTACCAGGGTATCTAAG, at a ratio 40:5:5:45:5). The primers were fused to Illumina adapter sequences (5′ TCGTCGGCAGCGTCAGATGTGTATAAGAGACAG for the forward primers and 5′ GTCTCGTGGGCTCGGAGATGTGTATAAGAGACAG for the reverse primers), according to manufacturer specifications. For fungi, we used primers targeting the ITS2 region, ITSF:5′CATCGATGAAGAACGCAG and ITSR:5′ TCCTSCGCTTATTGATATGC, also fused to Illumina adapters. The reactions were set up according to kit’s instructions and the PCR was performed in a BioRad iCycler instrument with the following conditions: 95 °C for 10 min, then 32 cycles of 95 °C for 30 seconds, 55 °C for 30 seconds, and 72 °C for 3 min, followed by fluorescence read. After completion of target sequence amplification, enzymatic reaction cleanup was performed at 37 °C for 15 minutes, followed by inactivation at 95 °C for 10 min. Amplicon libraries were then barcoded using the ZymoBIOMICS Index Primer Set’s A, B, and C in a 5-cycle real time PCR reaction, following the kit protocol. Barcoded amplicons were pooled and purified using the Zymo Select-a-Size DNA Clean & Concentrator MagBead kit. Paired end sequencing (2 × 300 bp) of pooled amplicon libraries was performed on an Illumina MiSeq instrument (Illumina, San Diego, CA).

### Sequence analyses

**Fig. 2:**
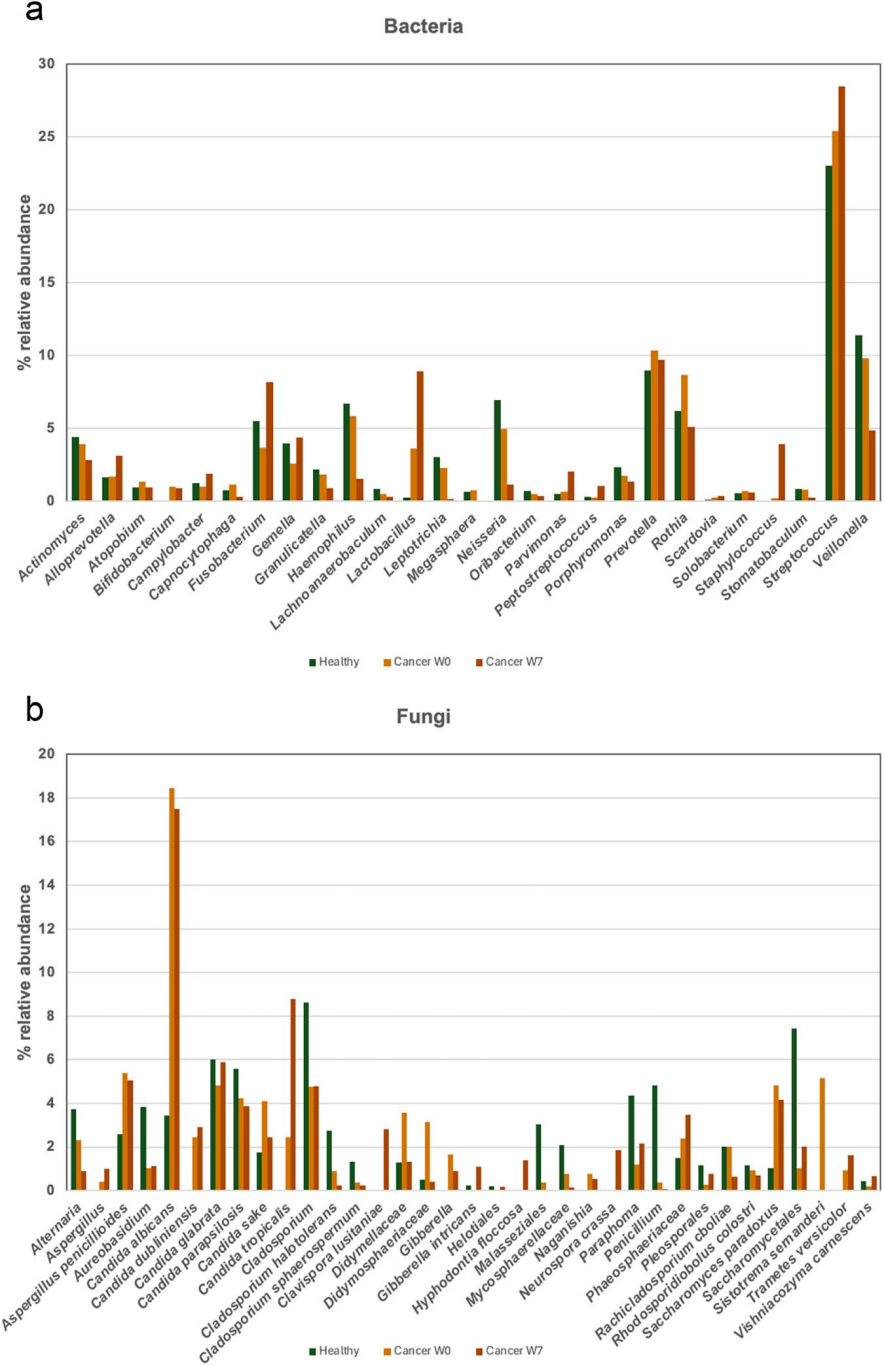
Microbiome diversity in healthy controls and cancer subjects. The most relatively abundant bacteria and fungi identified in healthy and cancer subjects were averaged across all sample types and timepoints for healthy subjects. For cancer subjects, data was averaged for weeks 0 and 7, respectively.

Forward and reverse sequence reads were demultiplexed on the Miseq instrument and assigned to the individual samples based on barcodes. The sequences were imported as paired fastq files into QIIME2^24^. The DADA2 plugin was used to trim, denoise, pair, purge chimeras and select amplicon sequence variants (ASVs), using the command “qiime dada2 denoise-paired”. Taxonomy was assigned using a pre-trained Naive Bayes classifier based on the SILVA 16S rRNA database (v138)^25^ trimmed to the 515 F/806 R region or the Unite (ITS) database^26^ for fungal sequences. Unassigned sequences, mitochondrial, and chloroplast sequences were removed. Summaries of the most relatively abundant genera and species of bacteria and fungi, averaged by subject category, are shown in Fig. 2. Sequence variant based richness and Shannon diversity were calculated within QIIME2 and tested for phenotype and sample type with ANOVA and Tukey HSD. Beta diversity (using Bray-Curtis dissimilarity distances) was calculated with the Phyloseq package in R and tested for phenotype and sample type with Permanova.

## Data Record

The amplicon sequence data and all the associated metadata have been deposited in the Sequence Reads Archive (SRA) of the National Canter for Biotechnology Information (NCBI) under the SRA Study SRP531770^27^ as part of BioProject PRJNA1158838. Metadata includes clinical information (cancer stage, mucositis, treatments) as well as patient/healthy controls demographics and ancillary data (nutrition, dentition, tobacco and alcohol use). That data is also provided in the Supplementary Table S1, S2.

## Technical Validation

**Fig. 3:**
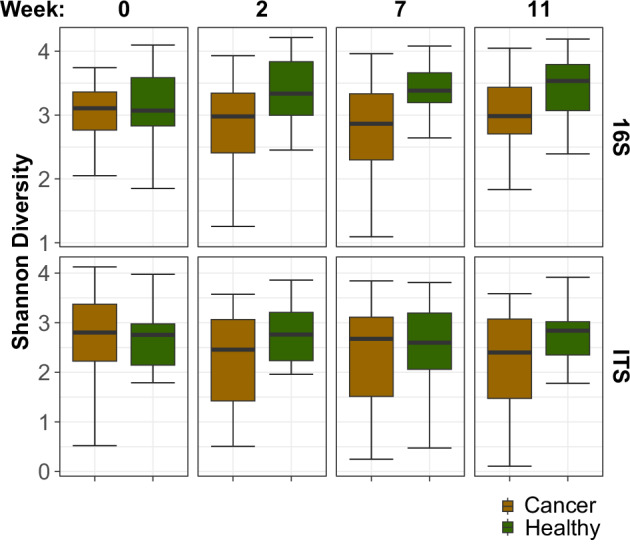
Microbiome alpha diversity. Shannon alpha diversity was calculated for healthy control and cancer cohorts for each sampling timepoint.

Technical validation steps were performed using standard best practices in microbial ecology. Negative control DNA extraction and amplicon sequencing were performed and supported the absence of reagents or handling-associated contamination. The forward and reverse fastq sequence reads were trimmed during the assembly process based on quality (>Q30). Error correction and chimera detection by the DADA software filtered out PCR and sequencing artifacts. The median number of raw sequences was 54,406 (16S) and 51,037 (ITS) per sample, respectively. Following read assembly and quality filtering, the datasets contained a median 35,562 (16S) and 22,794 (ITS) sequences per sample. The amplicons were assigned to bacterial and fungal taxa by applying the QIIME2 classify-sklearn algorithm to the reference SILVA and Unite databases. Across all samples and subjects, the 16S ASVs were assigned to 323 bacterial taxa and the ITS ASVs to 431 fungal taxa (Supplementary Table S3, S4). Microbial and fungal alpha diversity varied across sampling site but did not correlate significantly with subject health category or sampling time, although the diversity spread was higher in cancer subjects (Fig. 3). Permanova tests indicate that the community composition (beta diversity, for both bacterial and fungal) were different between the healthy and the cancer cohorts (p < 0.001) and, in the disease group, were also impacted by chemotherapy (p < 0.001), participation in the Galera trial (p < 0.001) the presence of mucositis (bacteria p < 0.001, fungi p < 0.005, respectively) or thrush (p < 0.001). No significant differences were associated with the type of cancer, sample collection site and lifestyle metadata.

## Usage Notes

A limitation of our study is the small cohort of subjects that were sampled relative to the many variables associated with oral cancer, different treatments, secondary complications and outcomes. The data we presented here could inform future studies to further address the complexity of oral cancer-microbiome-mucositis connections.

The raw sequence data (forward and reverse fastq sequences) can be downloaded from the NCBI SRA database. The following scripts can be used in QIIME2 to perform read processing and taxonomic assignments using the 16S rRNA and ITS references available in the QIIME2 repository. The output qza files can serve as input for a variety of statistical and diversity analyses in conjunction with other similar datasets from similar studies.

### 16S reads processing

# 16S processing for two different runs

qiime tools import --type ‘SampleData[PairedEndSequencesWithQuality]’ \

--input-path /16Sreads/ \

--output-path 16S_demux.qza \

--input-format CasavaOneEightSingleLanePerSampleDirFmt

qiime demux summarize \

--i-data 16S_demux.qza \

--o-visualization 16S_demux.qzv

qiime dada2 denoise-paired --i-demultiplexed-seqs. 16S_demux.qza \

--p-trim-left-f 19 \

--p-trim-left-r 19 \

--p-trunc-len-f 220 \

--p-trunc-len-r 220 \

--o-table 16S-dada2table.qza \

--o-representative-sequences 16S-rep-seqs.qza \

--o-denoising-stats 16S-denoising-stats.qza \

qiime metadata tabulate \

--m-input-file 16S-denoising-stats.qza \

--o-visualization 16S-stats.qzv

qiime feature-classifier classify-sklearn \

--i-classifier silva-132-99-515-806-nb-classifier.qza \

--i-reads 16S-rep-seqs.qza \

--o-classification 16S-taxonomy.qza \

qiime metadata tabulate \

--m-input-file 16S-taxonomy.qza \

--o-visualization 16S-taxonomy.qzv

### ITS reads processing

qiime tools import–type ‘SampleData[PairedEndSequencesWithQuality]’ \

--input-path /ITSreads/ \

--output-path ITS_demux.qza \

--input-format CasavaOneEightSingleLanePerSampleDirFmt

qiime demux summarize \

--i-data path ITS_demux.qza \

--o-visualization ITS_demux.qzv

qiime dada2 denoise-paired–i-demultiplexed-seqs ITS-demux.qza \

--p-trim-left-f 19 \

--p-trim-left-r 19 \

--p-trunc-len-f 220 \

--p-trunc-len-r 220 \

--o-table ITS-dada2table.qza \

--o-denoising-stats ITS-denoising-stats.qza \

qiime metadata tabulate \

--m-input-file ITS-denoising-stats.qza \

--o-visualization ITS-stats.qzv

qiime feature-classifier classify-sklearn \

--i-classifier unite-ver8-dynamic-classifier-07Nov2019.qza \

--i-reads ITS-rep-seqs.qza \

--o-classification ITS-taxonomy.qza \

## Supplementary information

Supplementary Table S1

Supplementary Table S2

Supplementary Table S3

Supplementary Table S4

## Data Availability

No custom code was used in this study. All sequence data analyses were performed using QIIME2 v.2020.8. That version and newer versions can be freely downloaded and installed from https://docs.qiime2.org/2024.5/install/.

## References

[1] Shankar, A. *et al*. Current Trends in Management of Oral Mucositis in Cancer Treatment. *Asian Pac J Cancer Prev* **18**, 2019–2026, 10.22034/APJCP.2017.18.8.2019 (2017). 28843216 10.22034/APJCP.2017.18.8.2019 PMC5697454

[2] Sant Ana, G., Normando, A. G. C., De Toledo, I., Dos Reis, P. E. D. & Guerra, E. N. S. Topical Treatment of Oral Mucositis in Cancer Patients: A Systematic Review of Randomized Clinical Trials. *Asian Pac J Cancer Prev* **21**, 1851–1866, 10.31557/APJCP.2020.21.7.1851 (2020). 32711408 10.31557/APJCP.2020.21.7.1851 PMC7573410

[3] San Valentin, E. M. D., Do, K. A., Yeung, S. J. & Reyes-Gibby, C. C. Attempts to Understand Oral Mucositis in Head and Neck Cancer Patients through Omics Studies: A Narrative Review. *Int J Mol Sci* **24**, 10.3390/ijms242316995 (2023). 10.3390/ijms242316995 PMC10706892 38069314

[4] Peterson, D. E., Bensadoun, R. J., Roila, F. & Group, E. G. W. Management of oral and gastrointestinal mucositis: ESMO Clinical Practice Guidelines. *Ann Oncol* **21** (Suppl 5), v261–265, 10.1093/annonc/mdq197 (2010). 20555094 10.1093/annonc/mdq197

[5] Elad, S. *et al*. MASCC/ISOO clinical practice guidelines for the management of mucositis secondary to cancer therapy. *Cancer* **126**, 4423–4431, 10.1002/cncr.33100 (2020). 32786044 10.1002/cncr.33100 PMC7540329

[6] Bowen, J. *et al*. The pathogenesis of mucositis: updated perspectives and emerging targets. *Support Care Cancer* **27**, 4023–4033, 10.1007/s00520-019-04893-z (2019). 31286231 10.1007/s00520-019-04893-z

[7] Abdalla-Aslan, R., Keegan, R., Zadik, Y., Yarom, N. & Elad, S. Recent advances in cancer therapy-associated oral mucositis. *Oral Dis*10.1111/odi.14999 (2024). 38968169 10.1111/odi.14999

[8] Stringer, A. M. & Logan, R. M. The role of oral flora in the development of chemotherapy-induced oral mucositis. *J Oral Pathol Med* **44**, 81–87, 10.1111/jop.12152 (2015). 24494824 10.1111/jop.12152

[9] Sixou, J. L., de Medeiros-Batista, O. & Bonnaure-Mallet, M. Modifications of the microflora of the oral cavity arising during immunosuppressive chemotherapy. *Eur J Cancer B Oral Oncol***32b**, 306–310, 10.1016/0964-1955(96)00006-1 (1996). 8944833 10.1016/0964-1955(96)00006-1

[10] Hong, B. Y. *et al*. Chemotherapy-induced oral mucositis is associated with detrimental bacterial dysbiosis. *Microbiome* **7**, 66, 10.1186/s40168-019-0679-5 (2019). 31018870 10.1186/s40168-019-0679-5 PMC6482518

[11] Zhang, L. *et al*. Influence of oral microbiome on longitudinal patterns of oral mucositis severity in patients with squamous cell carcinoma of the head and neck. *Cancer* **130**, 150–161, 10.1002/cncr.35001 (2024). 37688396 10.1002/cncr.35001 PMC10872366

[12] Vasconcelos, R. M. *et al*. Host-Microbiome Cross-talk in Oral Mucositis. *J Dent Res* **95**, 725–733, 10.1177/0022034516641890 (2016). 27053118 10.1177/0022034516641890 PMC4914867

[13] Mougeot, J. C., Stevens, C. B., Morton, D. S., Brennan, M. T. & Mougeot, F. B. Oral Microbiome and Cancer Therapy-Induced Oral Mucositis. *J Natl Cancer Inst Monogr* **2019**, 10.1093/jncimonographs/lgz002 (2019). 10.1093/jncimonographs/lgz002 31425594

[14] Fernandez Forne, A. *et al*. Influence of the microbiome on radiotherapy-induced oral mucositis and its management: A comprehensive review. *Oral Oncol* **144**, 106488, 10.1016/j.oraloncology.2023.106488 (2023). 37399707 10.1016/j.oraloncology.2023.106488

[15] Bruno, J. S. *et al*. From Pathogenesis to Intervention: The Importance of the Microbiome in Oral Mucositis. *Int J Mol Sci* **24**, 10.3390/ijms24098274 (2023). 10.3390/ijms24098274 PMC10179181 37175980

[16] Donnelly, J. P., Bellm, L. A., Epstein, J. B., Sonis, S. T. & Symonds, R. P. Antimicrobial therapy to prevent or treat oral mucositis. *Lancet Infect Dis* **3**, 405–412, 10.1016/s1473-3099(03)00668-6 (2003). 12837345 10.1016/s1473-3099(03)00668-6

[17] Min, Z., Yang, L., Hu, Y. & Huang, R. Oral microbiota dysbiosis accelerates the development and onset of mucositis and oral ulcers. *Front Microbiol* **14**, 1061032, 10.3389/fmicb.2023.1061032 (2023). 36846768 10.3389/fmicb.2023.1061032 PMC9948764

[18] Bolyen, E. *et al*. Reproducible, interactive, scalable and extensible microbiome data science using QIIME 2. *Nat Biotechnol* **37**, 852–857, 10.1038/s41587-019-0209-9 (2019). 31341288 10.1038/s41587-019-0209-9 PMC7015180

[19] Methe, B. A. *et al*. A framework for human microbiome research. *Nature* **486**, 10.1038/nature11209 (2012). 10.1038/nature11209 PMC3377744 22699610

[20] Sepich-Poore, G. D. *et al*. The microbiome and human cancer. *Science* **371**, 10.1126/science.abc4552 (2021). 10.1126/science.abc4552 PMC8767999 33766858

[21] Pasolli, E. *et al*. Extensive Unexplored Human Microbiome Diversity Revealed by Over 150,000 Genomes from Metagenomes Spanning Age, Geography, and Lifestyle. *Cell* **176**, 649–662 e620, 10.1016/j.cell.2019.01.001 (2019). 30661755 10.1016/j.cell.2019.01.001 PMC6349461

[22] CM, A. *et al*. ROMAN: Phase 3 trial of avasopasem manganese (GC4419) for severe oral mucositis (SOM) in patients receiving chemoradiotherapy (CRT) for locally advanced, nonmetastatic head and neck cancer (LAHNC). *J. Clin Oncol* **40** (2022).

[23] Miller, A. B., Hoogstraten, B., Staquet, M. & Winkler, A. Reporting results of cancer treatment. *Cancer* **47**, 207-214, 10.1002/1097-0142(19810101)47:1<207::aid-cncr2820470134>3.0.co;2-6 (1981). 10.1002/1097-0142(19810101)47:1<207::aid-cncr2820470134>3.0.co;2-6 7459811

[24] Estaki, M. *et al*. QIIME 2 Enables Comprehensive End-to-End Analysis of Diverse Microbiome Data and Comparative Studies with Publicly Available Data. *Curr Protoc Bioinformatics* **70**, e100, 10.1002/cpbi.100 (2020). 32343490 10.1002/cpbi.100 PMC9285460

[25] Quast, C. *et al*. The SILVA ribosomal RNA gene database project: improved data processing and web-based tools. *Nucleic Acids Res* **41**, D590–596, 10.1093/nar/gks1219 (2013). 23193283 10.1093/nar/gks1219 PMC3531112

[26] Abarenkov, K. *et al*. The UNITE database for molecular identification and taxonomic communication of fungi and other eukaryotes: sequences, taxa and classifications reconsidered. *Nucleic Acids Res* **52**, D791–D797, 10.1093/nar/gkad1039 (2024). 37953409 10.1093/nar/gkad1039 PMC10767974

[27] *NCBI Sequence Read Archive* https://identifiers.org/ncbi/insdc.sra:SRP531770.

[28] MB, A. *et al*. *AJCC Cancer Staging Manual, Eighth Edition*. (American College of Surgeons Springer, 2018).

